# Traumatic Life Events in Relation to Cognitive Flexibility: Moderating Role of the BDNF Val66Met Gene Polymorphism

**DOI:** 10.3389/fnbeh.2017.00241

**Published:** 2017-12-08

**Authors:** Robert L. Gabrys, Kaylyn Dixon, Hymie Anisman

**Affiliations:** Department of Neuroscience, Carleton University, Ottawa, ON, Canada

**Keywords:** BDNF Val66Met, traumatic events, early life stress, cognitive flexibility, set-shifting, Wisconsin Card Sorting Task

## Abstract

Cognitive flexibility plays an important role in an individual's ability to adapt to a continuously changing environment and is considered central to goal-oriented behavior. Accordingly, increasing attention has been devoted to understanding the factors, including genetic and early life experiences, which might contribute to individual differences in this ability. In the present investigation, we examined the contribution of the BDNF *Val66Met* polymorphism to cognitive flexibility, as assessed by set-shifting ability on the Wisconsin Card Sorting Task (WCST), and whether this polymorphism moderated the relation between trauma experiences (including type and timing of trauma occurrence) and cognitive flexibility. Among undergraduate students (*N* = 239), greater frequency of total traumas experienced prior to the age 5 was associated with greater difficulties in set-shifting (as indexed by more frequent perseverative errors on the WCST) among individuals carrying the *Met* allele of the BDNF polymorphism, but not those who were *Val* homozygotes. By contrast, total traumas experienced between the age of 6 to 12 and 13 to 18 were not related to set-shifting ability, and these relations were not moderated by BDNF genotype. Moreover, greater frequency of general traumas and emotional abuse was associated with set-shifting difficulties for both male and female *Met* allele carriers, but not *Val* homozygotes. In contrast, physical punishment was related to difficulties in set-shifting, but only among male *Met* carriers, an effect that was likely attributed to greater frequency of this form of trauma among males. The present findings suggest that the relationship between early life trauma and later-life cognitive flexibility might depend on the presence of the BDNF *Val66Met* polymorphism as well as the development stage at which the trauma has occurred. Moreover, the present investigation provides further understanding into the factors (i.e., genetic and early life experiences) that might be associated with individual differences in cognitive functioning and goal-directed behaviors, such as problem-solving and decision-making.

## Introduction

Cognitive flexibility comprises the ability to modify cognitive strategies (“sets”) in response to changing environmental demands, and is considered to be a central aspect of goal-oriented behaviors, such as creative problem-solving and multi-tasking (Ionescu, 2012). At the most fundamental level, this ability is the manifestation of several cognitive (executive) control processes, including attention, working memory, and response inhibition (Dajani and Uddin, 2015), and is governed by fronto-parietal and fronto-striatal neuronal circuits (Kehagia et al., 2010; Dajani and Uddin, 2015). Impaired cognitive flexibility is generally marked by perseverative responding to goal-irrelevant (or inappropriate) information, and has been observed in multiple psychiatric illnesses, including depressive disorders (Trivedi and Greer, 2014), obsessive compulsive disorder (Chamberlain et al., 2006), and schizophrenia (Thoma et al., 2007). Although cognitive flexibility has begun to receive increased attention with respect to its involvement in goal-oriented behavior and psychiatric illness, the factors which contribute to individual differences in this ability have yet to be fully explored.

Brain Derived Neurotrophic Factor (BDNF) plays an important role in the growth and differentiation of neurons, synaptic plasticity, and maintenance of neurons in adult life (Lewin and Barde, 1996), and has been implicated as a key neurobiological mediator of learning and memory processes (Kovalchuk et al., 2002; Egan et al., 2003; Cunha et al., 2010). Although relatively few studies have examined the significance of this neurotrophin to cognitive flexibility in humans, research in animals indicated that manipulation of BDNF signaling can influence performance on behavioral paradigms assessing this ability in rodents (Savitz et al., 2006; D'Amore et al., 2013; Sakata et al., 2013). Specifically, intra-striatal infusions of BDNF in mice facilitated strategy shifting by minimizing perseverative responding to a previously acquired strategy (D'Amore et al., 2013), whereas attenuation of activity-dependent BDNF expression in rats disrupted spatial memory reversal and contextual memory extinction (Sakata et al., 2013). In effect, it is possible that genes regulating the expression of BDNF might contribute to differences in cognitive flexibility.

A common polymorphism in the BDNF gene (*Val*66*Met*) produces a *Val* to *Met* amino acid substitution of the pro-BDNF sequence, which leads to disruptions of BDNF functioning (Egan et al., 2003). The *Met* allele has been related to reduced hippocampal and prefrontal cortical volume (Pezawas et al., 2004) as well as disturbances in learning and memory processes (Bath and Lee, 2006). Moreover, in comparison to *Val* homozygotes, individuals carrying the *Met* allele of the BDNF polymorphism reported greater rumination (Beevers et al., 2009), an emotion regulation strategy associated with reduced cognitive flexibility (Davis and Nolen-Hoeksema, 2000). Although no studies have examined the contribution of the BDNF *Val66Met* polymorphism to cognitive flexibility in healthy individuals, among those with bipolar disorder, *Met* carriers of this polymorphism committed more perseverative errors on the WCST (Rybakowski et al., 2003, 2006). In the present study, it was hypothesized that, relative to individuals with the *Val/Val* genotype, *Met* allele carriers of the BDNF polymorphism would display reduced cognitive flexibility, as assessed through difficulties in set-shifting on the WCST.

The BDNF *Val66Met* polymorphism has been shown to interact with early life stressors as well as current life events in predicting negative cognitive and emotional outcomes. For instance, among *Met* allele carriers, early life stressors were accompanied by decreased volume in the prefrontal cortex (PFC) and hippocampus as well as impaired working memory and elevated symptoms of depression and anxiety (Gatt et al., 2009). Likewise, in response to current or ongoing stressors, individuals who carried the *Met* allele reported more frequent rumination and symptoms of depression (Clasen et al., 2011). However, the potential moderating role of this BDNF polymorphism in the relation between stressful life events and cognitive flexibility has not been explored. This said, adolescents who experienced childhood trauma (physical abuse and neglect) committed more frequent perseverative, but not non-perseverative, errors on the WCST, in comparison to those who did not experience these events (Spann et al., 2012). Similarly, early life stressor exposure in rodents produced a selective deficit in set-shifting and reversal learning (Han et al., 2011; Baudin et al., 2012), which was accompanied by alterations in BDNF protein expression in the PFC and nucleus accumbens (Han et al., 2011). Given this latter finding, we hypothesized that greater frequency of trauma would be related to reduced cognitive flexibility, particularly among individuals carrying the *Met* allele of the BDNF polymorphism, or those with altered BDNF functioning.

Cognitive flexibility skills, as with executive functions in general, vary over the course of development (Dajani and Uddin, 2015; Buttelmann and Karbach, 2017). In particular, cognitive flexibility skills begin developing at age 4 (Zelzo, 2006; Dick, 2014), with a sharp increase in competence of these abilities occurring between 7 and 9 years of age (Chelune and Baer, 1986; Huizinga and van der Molen, 2007). By age 10, cognitive flexibility is typically fully developed (Chelune and Baer, 1986), although improvements in this ability can persist throughout adolescence and into adulthood (Anderson, 2002; Huizinga et al., 2006). Given these developmentally-related cognitive changes, in the present study, it was of interest to determine whether the relation between cognitive flexibility in adulthood and early life trauma varied across several developmental stages. To this end, we examined the relation between WCST performance in adults and traumas reported to have occurred between the age of 0 to 5, a period when cognitive flexibility is presumably immature (Dick, 2014), between the age of 6 to 12, in which cognitive flexibility skills develop rapidly (Chelune and Baer, 1986; Dajani and Uddin, 2015), and 13 to 18 years of age, a time when cognitive flexibility is thought to be fully developed (Dick, 2014; Dajani and Uddin, 2015). We hypothesized that the moderating role of the BDNF *Val66Met* polymorphism in the relation between frequency of trauma and diminished cognitive flexibility would be most pronounced during earlier developmental stages (i.e., 0 to 5 and 6 to 12 years of age), as this time is especially important for the development of cognitive flexibility (Chelune and Baer, 1986; Zelzo, 2006; Dick, 2014), relative to that seen later (at the 13 to 18 years of age).

The association between trauma and cognitive flexibility might also depend on the type of traumatic event (or stressor) encountered (Hurtubise and Howland, 2017). For instance, greater frequency of childhood physical abuse and neglect, but not emotional abuse or neglect, was associated with impaired set-shifting ability among adolescents (Spann et al., 2012). Thus, in the present investigation, we examined whether the relation between trauma and cognitive flexibility would vary across several additional forms of traumatic events, including general traumas (e.g., serious personal injuries or illness, death or serious illness of a loved one, family history of violence, mental illness, or alcohol/drug abuse), physical punishment, emotional abuse, and sexual abuse. Furthermore, it was previously reported that males and females differ in the types of stressors encountered (Kendler et al., 2001), including those experienced early in life (Meng and D'Arcy, 2016). In particular, it has consistently been reported that males were more likely to experience physical abuse and interpersonal violence (Thompson et al., 2004; Meng and D'Arcy, 2016), whereas females were more likely to encounter sexual abuse (Maikovich-Fong and Jaffee, 2010; Stoltenborgh et al., 2011; de Waal et al., 2017). It was also suggested that males and females might differ in their sensitivity to certain types of stressful events, including childhood abuse, with women generally reporting more negative outcomes than men (MacMillan et al., 2001; Thompson et al., 2004; Afifi et al., 2016; Meng and D'Arcy, 2016). Given these findings, we further examined whether the moderating role of the BDNF *Val66Met* polymorphism in the relation between type of trauma and cognitive flexibility varied among males and females. As females typically exhibited more negative outcomes following traumatic experiences (e.g., MacMillan et al., 2001), in the present investigation, it was hypothesized that females would display a greater disturbance in cognitive flexibility than would males. However, it was expected that females would be particularly affected by emotional and sexual abuse, whereas males would be affected to a greater extent by physical abuse. Moreover, it was hypothesized that these sex-specific relations would be most pronounced among *Met* allele carriers of the BDNF polymorphism than those who were *Val* allele homozygous.

## Material and methods

### Participants

The present study involved 239 (female: *n* = 147) Carleton University undergraduate students. Given that BDNF polymorphism frequency varies across cultural groups (e.g., Euro-Caucasian vs. Asian), all participants were of Euro-Caucasian decent, ranging in age from 17 to 31 (*M* = 19.38, *SD* = 3.37). None of the participants reported a neurological disorder or learning disability, 24 participants were currently taking anti-depressant and/or anti-anxiety medication, and 2 individuals were using the psychostimulant methylphenidate.

### Procedure

Once signed informed consent was obtained, participants provided a saliva sample for genotyping, while completing a series of questionnaires pertaining to demographics variables (e.g., age and sex), current levels of anxiety, and affective state. Once these questionnaires were finished, participants completed a computerized version of the WCST. This task was followed by a self-report measure of early life trauma. All procedures were approved by the Carleton University Ethics Committee for Psychological Research.

### Genotyping

Samples for genotyping were collected using Norgen collection kits (Norgen Biotek Corp., Thorold, Ontario Canada). Genomic DNA was extracted from the sample collection kits according to the manufacturer's instructions, and diluted to approximately equal concentration (20 ng/μL). Samples were sent for genotyping to McGill University and Génome Québec Innovation Center (Montreal, Canada). Using polymerase chain reaction (PCR), the DNA was amplified, and QIAXcel was used to determine amplification status. Shrimp alkaline phosphatase was used to remove all unincorporated deoxyribose nucleoside triphosphates (dNTPs). One probe per marker was used to do a single base extension and the product was desalted using 6mg of resin. The product was spotted on Agena BioScience 96-well chips using a Samsung Nanodispenser, and the chip read by a Mass Spectrometer. A manual analysis was done for each marker. Primer sequences were as follows:

BDNF Val^66^Met forward: ACGTTGGATGTACTGAGCATCACCCTGGA
BDNF Val^66^Met reverse: ACGTTGGATGGCTTGACATCATTGGCTGAC
BDNF Val^66^Met probe: TCCAACAGCTCTTCTATCA

The allele distribution for the BDNF Val66Met polymorphism was 157 *Val/Val*, 61 *Val/Met*, and 4 *Met/Met*, which met Hardy-Weinberg Equilibrium expectations, $\chi_{\left(1\right)}^{2}$ =.484, *p* = 0.486. Due to the relative infrequency of *Met* homozygotes, we collapsed across *Val/Met* and *Met/Met* carriers, as done in previous studies (Caldwell et al., 2013). A total of 17 individuals were excluded from the genotype analyses because we were either unable to determine a genotype from the samples provided or the individual chose not to provide a saliva sample.

### Cognitive flexibility: set-shifting

Cognitive flexibility was determined using a computerized version of the WCST provided by the Psychology Experiment Building Language (PEBL) version 0.14, referred to as Berg's Card Sorting Task (BCST) (Mueller and Piper, 2014). The BCST consists of a 128 card deck with each card containing a different combination of one of four shapes, colors, and quantities. Four key cards are displayed at the top of the screen as a guide to help determine to which of the four stacks the deck's up-card is sorted. The deck is revealed one card at a time, and the visible card is matched to key cards depending on the particular rule (unknown to the participant) for a given set. After ten cards have been successfully matched (i.e., the participant has acquired the rule for the first attentional set), the set is completed and the sorting rule changes (also unknown to the participant). The new rule must be discovered using trial and error and through feedback received after each card is sorted. After a card is sorted, the participant is provided with feedback regarding whether it was sorted correctly (i.e., according to the current rule). This process continues until the participant either sorts all 128 cards, or until the participant successfully completes 9 sets/categories (for more information see Fox et al., 2013). The BCST takes approximately 10 min to complete.

The primary measures of the BCST are the type of errors the individual makes. *Perseverative errors* occur when the individual continues to sort cards according to a previously, but no longer, relevant or correct sorting rule. These types of errors were the central measure of reduced cognitive flexibility within this behavioral paradigm. In the present study, perseverative errors were computed according to procedures described by Heaton et al. (1993). *Non-perseverative errors*, by contrast, refer to all other errors. Among the non-perseverative errors are *failures to maintain set*, which refers to selecting an incorrect card once a sorting rule has been learned (i.e., switching after the fifth correctly sorted card). Failures to maintain a set are thought to represent the individual's distractibility or difficulties maintaining information in working memory (Barceló and Knight, 2002). Finally, the BCST also assesses *trials to first category*, which refers to the speed of which an individual acquires an attentional set.

### Measures

#### Traumatic life events

The frequency of traumatic life events was assessed using a modified version of the Early Trauma Inventory—Short Report (ETI-SR; Bremner et al., 2007). The ETI-SR is a 27-item self-report questionnaire assessing various types of early life events, including (i) general traumas (e.g., serious personal injuries or illness, death or serious illness of a parent, sibling, or friend, family history of violence, mental illness, or alcohol/drug abuse), (ii) physical punishment, (iii) emotional abuse, and (iv) sexual abuse. In the present study, for each item, participants were asked to indicate the frequency that each event occurred, from 0 (never) to 5 (more than 10 times), at 4 different age ranges (subscales), including 0 to 5, 6 to 12, 13 to 18, and after the age of 18. To generate a total frequency score for trauma the occurred between the age of 0 to 5, we summed across all 27 items (e.g., all types of trauma). This procedure was repeated for traumas that occurred between the age of 6 to 12 and 13 to 18. In the present study, we did not use the “traumas after 18” category as approximately 50% of the study sample was 18 years old or younger. Furthermore, to determine a total frequency for (i) *general traumas*, (ii) *physical punishment*, (iii) *emotional abuse*, and (iv) *sexual abuse*, we summed across all age subscales (i.e., 0 to 5, 6 to 12, 13 to 18, and after 18) for all items relevant to each type of trauma experience.

Table 1 displays the frequency of traumas that occurred between the age of 0 to 5, 6 to 12, and 13 to 18 as well as the frequency of different types of trauma among male and female participants in the present study. As shown in Table 1, males reported greater frequency of physical punishment than females, *t*_(162.91)_ = 4.06, *p* < 0.001, but no sex differences were observed for the frequency of general traumas, emotional abuse, or sexual abuse, or in the occurrence of total traumas experienced between the age of 0 to 5, 6 to 12, or 13 to 18 (*p*'s > 0.05).

**Table 1:** Mean (*SD*) sex differences in the timing and frequency of traumatic events.

	**Total trauma 0–5 years of age**	**Total trauma 6–12 years of age**	**Total trauma 13–18 years of age**	**General traumas**	**Physical punishment**	**Emotional abuse**	**Sexual abuse**
Males	3.73(5.76)	10.49(10.02)	15.51(10.91)	9.91(8.92)	11.50(11.50)^***^	14.70(16.71)	1.96(3.43)
Females	2.98(5.20)	10.02(11.27)	15.04(12.88)	11.39(11.39)	5.73(9.25)^***^	15.60(16.62)	2.90(4.45)

*** *p < 0.001*.

#### Anxiety

State anxiety prior to the WCST was assessed using the 20-item Spielberger State Trait Anxiety Inventory (STAI) (Spielberger et al., 1983). Items for this measure ranged from 1 (not at all) to 4 (very much), where higher scores indicated greater state anxiety. Total scores were obtained by summing across all items (α = 0.95) (*M* = 35.03, *SD* = 9.32).

#### Affective state

The 20-item Positive and Negative Affect Schedule (PANAS; Watson et al., 1988) was used to assess positive and negative affect prior to the WCST. Responses ranged on a six-point scale from 1 (very slightly or not at all) to 5 (extremely). Positive (*M* = 28.38, *S.D*. = 7.54) and negative (*M* = 14.50, *SD* = 14.15) affect scores were computed by summing across all 10 relevant items for each subscale. Cronbach's alphas were: positive (0.85) and negative (0.90).

### Statistical analyses

Statistical analyses were performed using SPSS for Windows 18.0 (SPSS Science, Chicago, IL). Pearson's correlation coefficients were determined between current mood state (i.e., state anxiety and positive and negative affect) and WCST performance indices. An independent samples *t*-test was used to examine whether individuals currently taking psycho-active medication differed from those who were not on WCST performance. A MANOVA was used to examine the contribution of BDNF genotype and Sex to performance indices of the WCST. Moderation analyses were carried out using the PROCESS add-on to SPSS provided by Hayes (2013). Specifically, using Model 2 in PROCESS, unstandardized frequency of Trauma scores were entered as the X (independent) variable, BDNF genotype as M1 (first moderator), Sex as M2 (second moderator), and perseverative errors (or other WCST performance indices) and the Y (outcome variable). Significant three-way interactions were followed up and graphed using the coefficients provided by Model 2 PROCESS output, whereas significant two-way interactions (e.g., when sex differences did not significantly contribute to the model) were followed up using Model 1.

## Results

Performance on the WCST, including the frequency of perseverative and non-preservative errors, failures to maintain set, and number of trials required to learn the first category, was not related to state anxiety, positive affect, or negative affect. Individuals who were currently taking psychoactive medications (*n* = 26) did not differ on any of the WCST performance indices. A MANOVA revealed no significant Genotype x Sex interaction on WCST performance. However, this analysis indicated that, in comparison to *Val* homozygotes (*M* = 10.84, *S.D*. = 4.14), *Met* (*M* = 12.41, *S.D*. = 5.85) allele carriers committed more frequent perseverative errors, *F*_(1, 226)_ = 6.36, *p* = 0.01, but did not differ on any other WCST indices. Additionally, males committed more perseverative errors, *F*_(1, 226)_ = 4.23, *p* < 0.05, and took more trials to learn the first sorting category, *F*_(1, 226)_ = 4.25, *p* < 0.05, than females.

### Timing of trauma in relation to cognitive flexibility: moderating role of BDNF genotype

**Figure 1:**
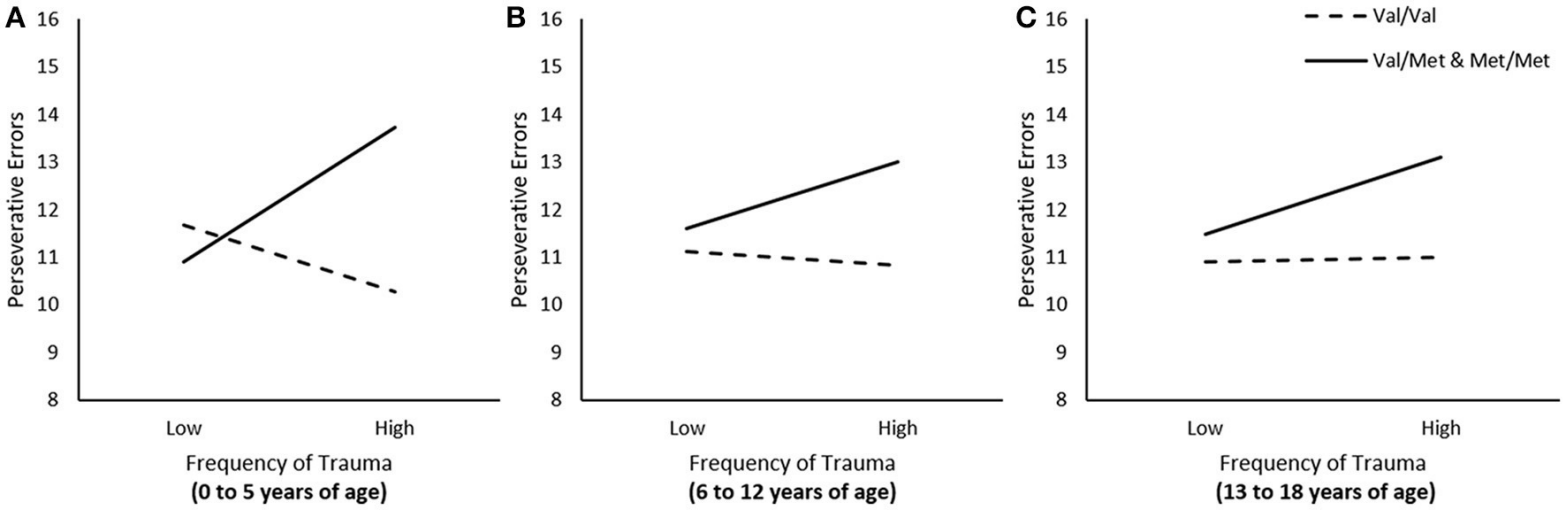
The moderating role of the BDNF *Val66Met* polymorphism in the relationship between total traumas between the age of **(A)** 0–5, **(B)** 6–13, and **(C)** 13–18 on frequency of perseverative errors on the WCST. Low frequency of total traumas = 1 *SD*. below the mean, high frequency of total traumas = 1 *SD*. above the mean.

We examined whether frequency of traumas experienced at different developmental stages (i.e., 0 to 5, 6 to 12, and 13 to 18 years of age) was associated with frequency of perseverative errors, and whether these relations were moderated by the BDNF genotype. In these regression analyses, when examining the relation between trauma experienced during one time frame (e.g., 0 to 5 years of age) and perseverative errors, we controlled for traumas that were encountered during other times (e.g., 6 to 12 and 13 to 18). The relation between frequency of total traumas that occurred between the age of 0 to 5 was moderated by BDNF genotype, Δ*R*^2^ = 0.04, *F*_(1, 222)_ = 9.36, *p* < 0.01. As illustrated in Figure 1A, simple slopes for this interaction indicated that greater frequency of traumas between the age of 0 to 5 was associated with more perseverative errors among individuals carrying the *Met* allele, *b* = 0.26, *t* = 2.30, *p* < 0.05, but not among *Val* homozygotes, *b* = −0.12, *t* = −1.34, *p* > 0.05. The frequency of total traumas that occurred between the age of 6 to 12 and between 13 to 18 were not moderated by BDNF genotype, Δ*R*^2^ = 0.01, *F*_(1, 222)_ = 1.60, 1.47, *p's* > 0.05, respectively (Figures 1B,C).

In addition to set-shifting performance (i.e., perseverative errors), we repeated these regression analyses to determine whether BDNF genotype moderated the relationship between traumas encountered at different developmental stages and other WCST performance indices. In this regard, traumas occurring between 0 to 5, 6 to 12, or 13 to 18 years of age were not related to non-perseverative errors, including the ability to acquire (i.e., trials to first category) and maintain (i.e., failures to maintain set) an attentional set, and these relations were not moderated by BDNF genotype.

### Type of trauma in relation to cognitive flexibility: moderating role of BDNF genotype

**Figure 2:**
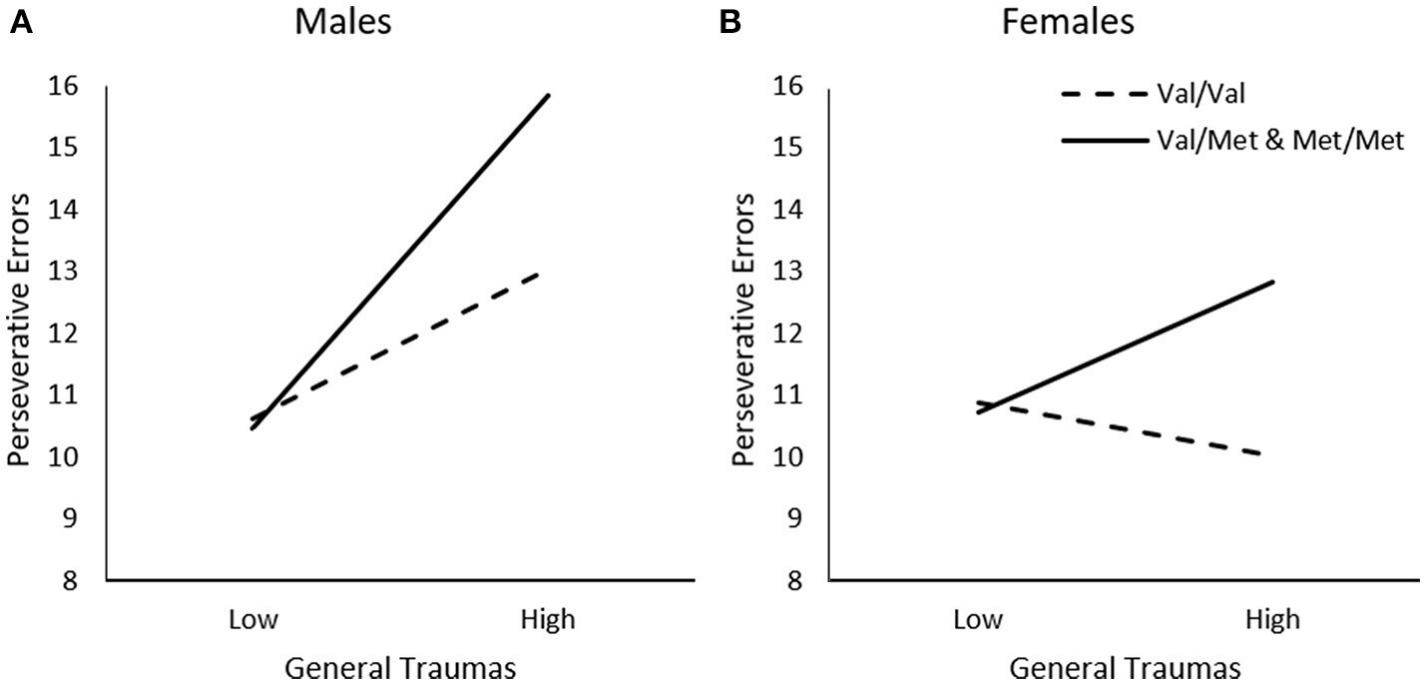
The moderating role of the BDNF Val66Met polymorphism in the relationship between frequency of general traumas and frequency of perseverative errors on the WCST among **(A)** males and **(B)** females. Low frequency of general traumas = 1 *SD*. below the mean, high frequency of general traumas = 1 *SD*. above the mean.

In addition to examining the relationship between timing of traumas, we examined whether the relationship between trauma and frequency of perseverative errors varied across different types of events, and whether this was moderated by the BDNF genotype and sex differences. When considering the frequency of General Traumas, Genotype, and Sex in relation to perseverative errors, a General Traumas x Genotype, Δ*R*^2^ = 0.02, *F*_(1, 226)_ = 5.75, *p* < 0.05, General Traumas × Sex, Δ*R*^2^ = 0.02, *F*_(1, 226)_ = 5.69, *p* < 0.05, and General Traumas × Genotype × Sex interaction, Δ*R*^2^ = 0.05, *F*_(1, 226)_ = 6.11, *p* < 0.01, was observed. As illustrated in Figure 2A, among males, greater frequency of general traumas was modestly, but not significantly, associated with more frequent perseverative errors among *Val* homozygotes, *b* = 0.11, *t* = 1.83, *p* = 0.07, and this relationship was much stronger for those carrying a *Met* allele, *b* = 0.26, *t* = 4.09, *p* < 0.001. In contrast, among females, greater frequency of general traumas was not related to perseverative errors among *Val* homozygotes, *b* = −0.04, *t* = −0.95, *p* > 0.05, and only modestly related among females carrying the *Met* allele, *b* = 0.10, *t* = 2.08, *p* < 0.05 (Figure 2B).

**Figure 3:**
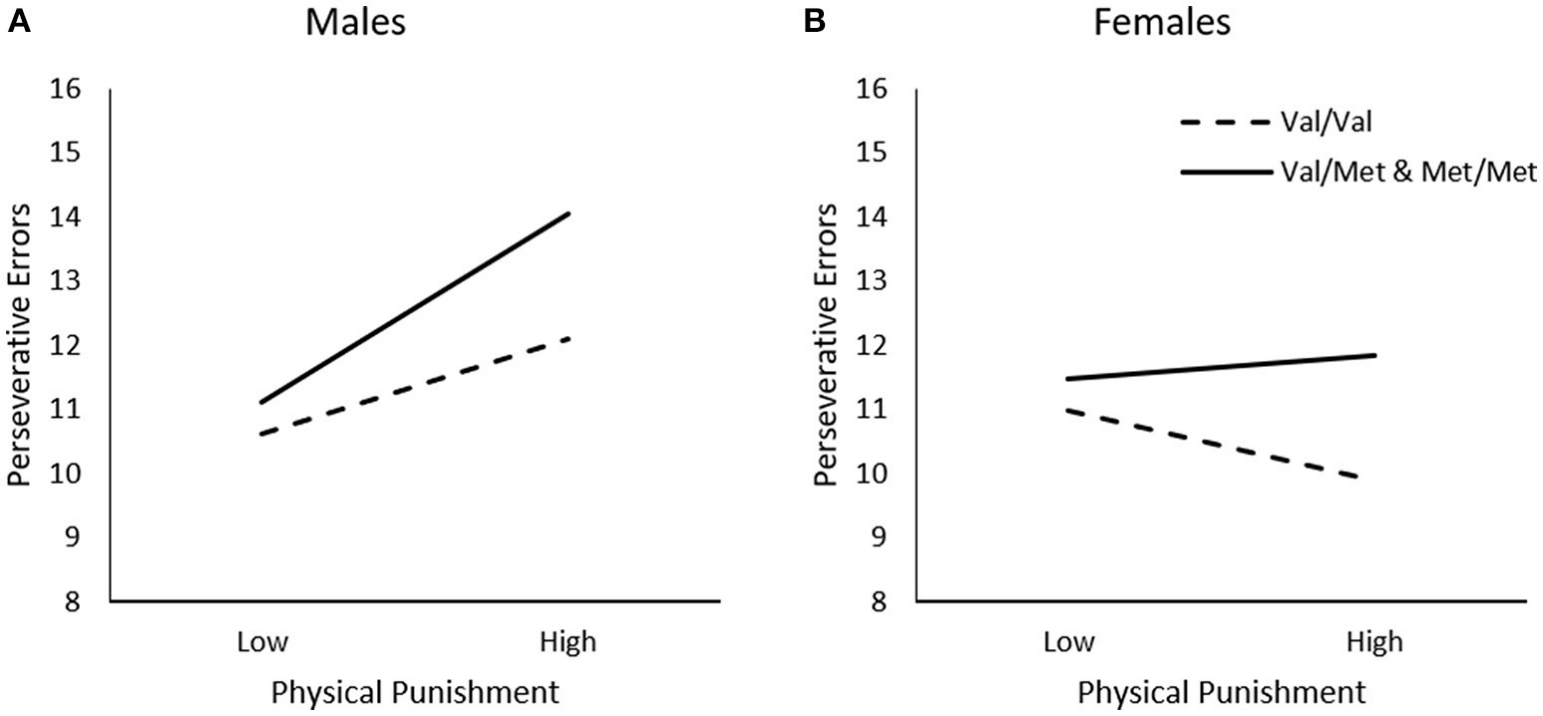
The moderating role of the BDNF Val66Met polymorphism in the relationship between frequency of physical punishment and frequency of perseverative errors on the WCST among **(A)** males and **(B)** females. Low frequency of physical punishment = 1 *SD*. below the mean, high frequency of physical punishment = 1 *SD*. above the mean.

In contrast to the findings observed for general traumas, perseverative errors varied as a function of the Physical Punishment × Sex interaction, Δ*R*^2^ = 0.02, *F*_(1, 226)_ = 3.82, *p* = 0.05, and the Physical Punishment x Genotype × Sex interaction was just shy of statistical significance, Δ*R*^2^ = 0.02, *F*_(1, 226)_ = 2.85, *p* = 0.06. As indicated by simple slopes analyses for the three-way interaction (Figure 3A), greater frequency of physical punishment was related to more perseverative errors among male *Met* carriers, *b* = 0.14, *t* = 2.54, *p* = 0.01, but not among *Val* homozygotes, *b* = 0.07, *t* = 1.33, *p* > 0.05. Among females, by contrast, physical punishment was not related to the frequency of perseverative errors among *Met* carriers, *b* = 0.02, *t* = 0.29, *p* > 0.05, or those who were homozygous for the *Val* allele, *b* = −0.05, *t* = −1.06, *p* > 0.05 (Figure 3B).

**Figure 4:**
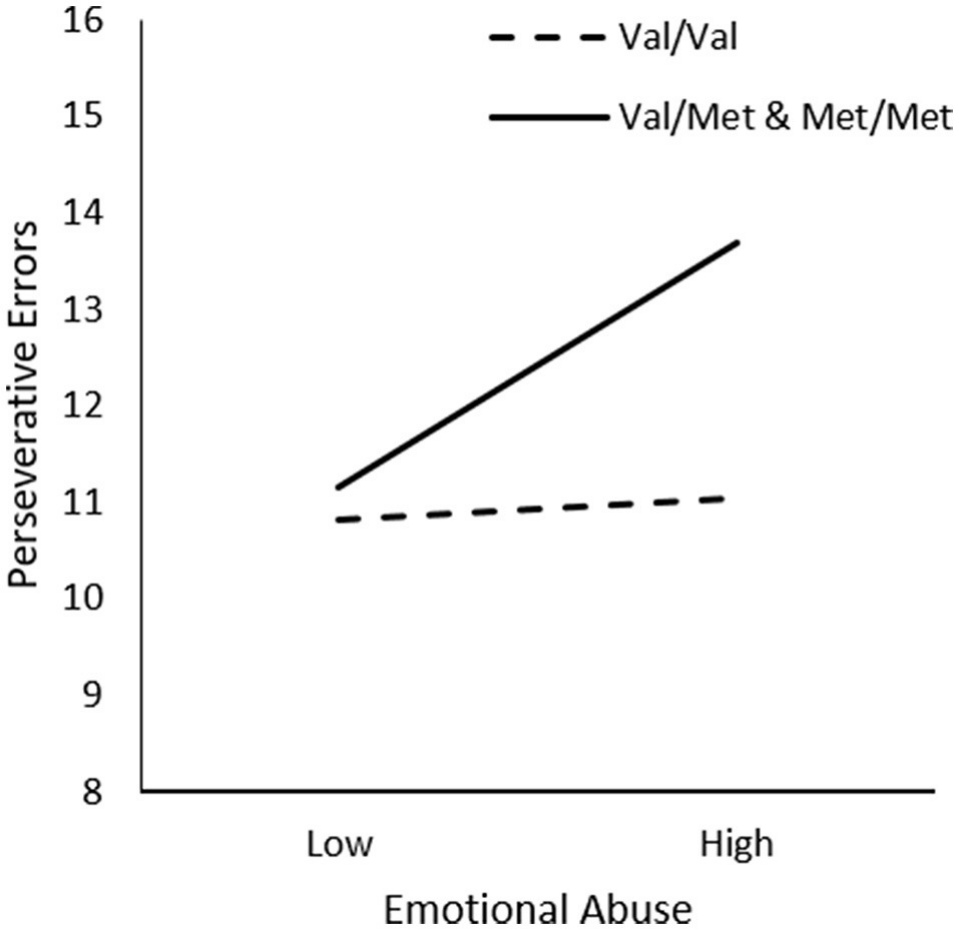
The moderating role of the BDNF Val66Met polymorphism in the relationship between frequency of emotional abuse and frequency of perseverative errors on the WCST. Low frequency of emotional abuse = 1 *SD*. below the mean, high frequency of emotional abuse = 1 *SD*. above the mean.

In examining the relationship between Emotional Abuse, Genotype, and Sex on frequency of perseverative errors, we did not observe significant interactions between Emotional Abuse x Genotype, Δ*R*^2^ = 0.01, *F*_(1, 226)_ = 2.76, *p* = 0.10 or Emotional Abuse x Genotype x Sex, Δ*R*^2^ = 0.02, *F*_(1, 226)_ = 1.99, *p* = 0.14. However, as shown in Table 1, in comparison to other forms of trauma, considerable inter-individual variability was apparent in the frequency of emotional abuse in the present sample, which might have contributed to the lack of statistical significance concerning this form of abuse. Given this possibility and *a priori* hypotheses, we proceeded with follow-up simple slopes analyses to determine whether perseverative errors varied as a function of emotional abuse and BDNF genotype. As shown in Figure 4, simple slopes for the two-way interaction indicated that emotional abuse was related to more perseverative errors for male and female *Met* carries, *b* = 0.08, *t* = 2.26, *p* < 0.05, but not for *Val* allele homozygotes, *b* = 0.01, *t* = 0.28, *p* > 0.05.

Finally, we did not observe a Sexual Abuse x Genotype, Sexual Abuse x Sex, or a Sexual Abuse x Genotype x Sex interaction in relation to the frequency of perseverative errors, Δ*R*^2^ = 0.00, *F*_(1, 226)_ =.03, *p* > 0.05. As indicated earlier, these findings might be due to the relative infrequency of sexual abuse reported by the present study sample.

Once again, we repeated these regression analyses to determine whether BDNF genotype moderated the relationship between different types of trauma and other WCST performance indices. In this regard, general traumas, physical punishment, emotional abuse, and sexual abuse were not related to non-perseverative errors, including the ability to acquire (i.e., trials to first category) and maintain (i.e., failures to maintain set) an attentional set, and BDNF genotype did moderate any of these relations.

## Discussion

The *Met* allele of the BDNF *Val66Met* polymorphism (RS6265) has been associated with disrupted BDNF functioning, decreased volume in prefrontal cortical and hippocampal regions (Pezawas et al., 2004), diminished neuroplasticity (Lamb et al., 2015), and impaired learning and memory processes (Bath and Lee, 2006). In the present investigation, the presence of the *Met* allele of this polymorphism was related to reduced cognitive flexibility, as reflected through difficulties in set-shifting (i.e., more frequent perseverative errors on the WCST). In fact, this behavioral profile among *Met* carriers was unique to perseverative response tendencies as individuals with this genotype did not differ from *Val* homozygotes in the frequency of non-perseverative errors, including the ability to acquire (i.e., trials required to learn the first sorting category) or maintain (i.e., failures to maintain set) an attentional set. These findings are reminiscent of those observed in rodents, wherein selective disruption of activity-dependent BDNF protein expression (i.e., in BDNF mutant rats) did not affect working, spatial, or fear memory, but contributed to pronounced perseverative responding, including impairments of reversal learning in a spatial memory task as well as in extinction of fear memory (Sakata et al., 2013). Thus, the *Met* allele of the BDNF gene (or disruptions in BDNF functioning) seems not to influence the ability to acquire or maintain a cognitive set (or behavioral strategy), but might specifically disrupt the ability to shift attention away from a previously, but no longer, appropriate behavioral strategy and toward one that is newly effective.

Stressful events, including those experienced early in life, can have a profound impact on PFC structure and functioning (Lupien et al., 2009; McEwen and Morrison, 2013; Arnsten et al., 2015), and it has become increasingly evident that these experiences can result in disturbed cognitive flexibility (Hurtubise and Howland, 2017). For instance, when assessed in adolescence, perseverative errors on the WCST, but not non-perseverative, were more frequent among individuals who had experienced childhood trauma (Spann et al., 2012). Similarly, in rodents, maternal deprivation (Baudin et al., 2012) and early social isolation (Han et al., 2011), produced a selective deficit in set-shifting and reversal learning, but not spatial learning, and this effect was accompanied by altered BDNF protein expression in the PFC and nucleus accumbens (Han et al., 2011). Consistent with these findings, in the present investigation, greater frequency of total traumas was associated with difficulties in set-shifting, but only among individuals carrying the *Met* allele of the BDNF polymorphism. Interestingly, however, this relationship was most pronounced among individuals who experienced relatively high levels of trauma prior to the age of 5. After this age (6 to 12 and 13 to 18 years of age), the moderating effects of the BDNF polymorphism in the relation between trauma frequency and set-shifting performance was progressively less notable and not statistically significant. Once again, these relations were unique to set-shifting ability as, regardless of development stage, traumas were not associated with the ability to acquire or maintain a set, nor were these relationships moderated by BDNF genotype. These findings are in keeping with the view that the skills necessary for cognitive flexibility begin to develop at an early age (i.e., approximately the age of 4), and are generally fully developed by about age 10 (Chelune and Baer, 1986; Zelzo, 2006; Huizinga and van der Molen, 2007; Buttelmann and Karbach, 2017). Thus, it is possible that trauma experienced particularly early in childhood might be most strongly accompanied by diminished cognitive flexibility later in adulthood.

These present findings are consistent with animal data indicating that stressors experienced early in life produced a selective impairment in set-shifting (Baudin et al., 2012; Hurtubise and Howland, 2017), and studies in humans which have investigated the moderating role of the BDNF *Val66Met* in the relationship between early life stressors and later-life negative cognitive, affective, biological outcomes. In particular, among *Met* allele carriers, early life stressors were accompanied by decreased volume in the PFC and hippocampus as well as impaired working memory and elevated symptoms of depression and anxiety (Gatt et al., 2009). Additionally, among individuals with the BDNF *Met* allele, high levels of childhood trauma were associated with lower levels of BDNF mRNA levels, diminished hippocampal subfield volumes, and reduced cognitive functioning (Aas et al., 2013). This is not to say that the moderating role of the BDNF *Val66Met* polymorphism in the relation between stressors (including trauma) and negative outcomes is restricted to early developmental periods. In this regard, in response to ongoing stressors, individuals who carried the *Met* allele reported more frequent rumination and symptoms of depression (Clasen et al., 2011). As will be recalled, although cognitive flexibility skills sharply increase early in life, improvements in these skills can persist throughout adolescence and into adulthood, peaking between the ages of 21–30 (Cepeda et al., 2001; Anderson, 2002; Huizinga et al., 2006; Huizinga and van der Molen, 2007). It is possible that the relation between current stressors and cognitive flexibility might also be influenced by the BDNF *Val66Met* polymorphism.

As varied stressors can have diverse effects on brain neurochemical processes and behavioral outcomes (Anisman et al., 2008), we examined whether the moderating effects of the BNDF *Val66Met* polymorphism in the relation between trauma and cognitive flexibility would vary across different traumatic events. Moreover, given that males and females might differ in the frequency and sensitivity to different forms of stressful and traumatic events (Thompson et al., 2004; Maikovich-Fong and Jaffee, 2010; Afifi et al., 2016; Meng and D'Arcy, 2016), we also considered sex differences in these relationships. In the present study, several forms of trauma, other than sexual abuse, were related to reduced set-shifting (but not the ability to acquire or maintain a set) among *Met* allele carriers, but not *Val* homozygotes. The strength of these associations, however, varied slightly between males and females across type of trauma. Among *Met* allele carriers, general traumas appeared to have the strongest relationship to reduced set-shifting, especially among males. As will be recalled, the general traumas subscale of the ETI-SR is comprised of various non-abuse related traumas, including serious personal injuries or illness, death or serious illness of a parent, sibling, or friend, family history of violence, mental illness, or alcohol/drug abuse. It is difficult to discern which of these traumas uniquely accounted for the observed effects. Indeed, it is possible that the cumulative actions of all these general traumas, coupled with the *Met* allele of the BDNF polymorphism, was most aligned with the poorer set-shifting performance.

It is not entirely clear why the association between general traumas and set-shifting performance was stronger among males. It has been reported that males and females differ in the types of negative outcomes that accompany certain traumas (Thompson et al., 2004; Maikovich-Fong and Jaffee, 2010; Meng and D'Arcy, 2016), but it is uncertain whether this is linked to specific hormonal factors. This said, although the performance difference between males and females was statistically significant, it is not clear that the sex-specific difference in the relation between general traumas and set-shifting performance was meaningful. This is underscored by the fact that among both male and female *Met* carriers, more frequent general traumas were associated with diminished set-shifting performance. It is also possible that the sex differences were simply a reflection of the greater proficiency of females in behavioral tasks assessing cognitive flexibility, particularly the WCST (Boone et al., 1993; Aly et al., 2015).

In contrast to the relations observed concerning general traumas, physical punishment was associated with diminished set-shifting, but only among male *Met* allele carriers. This effect was likely attributable to the greater frequency of physical punishment reported by males in the present study. Although it is possible that a history of physical punishment is associated with more detrimental effects on cognitive functioning in males than females, but there does not appear to be clear and consistent evidence to support this assumption. In contrast to physical punishment, in the present study, emotional abuse was associated with diminished set-shifting performance equally in male and female *Met* allele carriers, whereas sexual abuse was not related to set-shifting ability, regardless of BDNF genotype. However, a detailed analysis of the potential moderators between sexual abuse and cognitive flexibility was not feasible given that this form of trauma was infrequently reported.

Taken together, the present findings suggest that the relationship between greater frequency of trauma and diminished cognitive flexibility might be most pronounced among individuals carrying the BDNF *Met* allele, or those with altered BDNF functioning. Moreover, among individuals carrying the *Met* allele, reduced cognitive flexibility seemed to be most strongly tied to trauma experienced relatively early in life (e.g., prior to the age of 5). That said, the present data do not speak to the potential role of the BDNF *Val66Met* polymorphism in moderating the relation between current stressors and cognitive flexibility nor that of individuals who encountered both early life and adult trauma. In contrast to our hypotheses, the present data did not provide clear evidence that the association between trauma and cognitive flexibility (set-shifting performance) would vary based on the type of trauma experienced, and that this would be moderated by the BDNF polymorphism. Although slight differences across traumas were observed at least among *Met* allele carriers, various forms of trauma might be associated with diminished cognitive flexibility, irrespective of the form of the trauma. Once again, rather than any single form of trauma being uniquely related to performance disturbances, cumulative trauma experiences are most likely predictive of diminished cognitive flexibility, especially among *Met* allele carriers. Finally, although we did observe sex differences in the relationship between trauma type, the BDNF polymorphism, and cognitive flexibility, these differences appeared to be relatively small, and their meaningfulness is uncertain.

Several limitations should be considered regarding the findings of the present study. The assessment of early life trauma was determined through a self-report measure, which might have been susceptible to memory biases and difficulties recalling early life events. Moreover, given that the frequency of gene polymorphisms varies across ethnic groups, and that their influence on behavior might vary by ethnicity, the present sample included only Euro-Caucasian individuals. Thus, the present findings might not be generalizable to other ethnicities. Nonetheless, the present findings suggest that the relationship between early life trauma and later-life cognitive flexibility might depend on the presence of the BDNF *Val66Met* polymorphism as well as the development stage at which the trauma has occurred. Moreover, the present findings provide further understanding into the factors (i.e., genetic and early life experiences) that might be associated with individual differences in the ability to adapt to continuous changing environments and goal-directed behaviors, such as problem-solving and decision-making.

## Author contributions

RG, KD, and HA contributed equally to the conception and study design, data acquisition, analysis, and interpretation as well as drafting, revising, and final approval of the present manuscript.

### Conflict of interest statement

The authors declare that the research was conducted in the absence of any commercial or financial relationships that could be construed as a potential conflict of interest.

## References

[B1] Aas M. Haukvik U. K. Djurovic S. Bergmann Ø. Athanasiu L. Tesli M. S.. (2013). BDNF val66met modulates the association between childhood trauma, cognitive and brain abnormalities in psychoses. Prog. Neuropsychopharmacol. Biol. Psychiatry. 46, 181–188. 10.1016/j.pnpbp.2013.07.008 23876786

[B2] Afifi T. O. MacMillan H. L. Boyle M. Cheung K. Taillieu T. Turner S.. (2016). Child abuse and physical health in adulthood. Health Rep. 27, 10–18. 26983007

[B3] Aly H. Salama H. Ibrahim S. El-Shestawy H. (2015). Sex differences in cognitive dysfunction among bipolar disorder patients. Egypt. J. Psychiatry 36, 1–8. 10.4103/1110-1105.153766

[B4] Anderson P. (2002). Assessment and development of executive function (EF) during childhood. Child Neuropsychol. 8, 71–82. 10.1076/chin.8.2.71.8724 12638061

[B5] Anisman H. Merali Z. Hayley S. (2008). Neurotransmitter, peptide and cytokine processes in relation to depressive disorder: comorbidity between depression and neurodegenerative disorders. Prog. Neurobiol. 85, 1–74. 10.1016/j.pneurobio.2008.01.004 18346832

[B6] Arnsten A. F. Raskind M. A. Taylor F. B. Connor D. F. (2015). The effects of stress exposure on prefrontal cortex: translating basic research into successful treatments for post-traumatic stress disorder. Neurobiol. Stress 1, 89–99. 10.1016/j.ynstr.2014.10.002 25436222 PMC4244027

[B7] Barceló F. Knight R. T. (2002). Both random and perseverative errors underlie WCST deficits in prefrontal patients. Neuropsychologia 40, 349–356. 10.1016/S0028-3932(01)00110-5 11684168

[B8] Bath K. G. Lee F. S. (2006). Variant BDNF (Val66Met) impact on brain structure and function. Cogn. Affect. Behav. Neurosci. 6, 79–85. 10.3758/CABN.6.1.79 16869232

[B9] Baudin A. Blot K. Verney C. Estevez L. Santamaria J. Gressens P.. (2012). Maternal deprivation induces deficits in temporal memory and cognitive flexibility and exaggerates synaptic plasticity in the rat medial prefrontal cortex. Neurobiol. Learn. Mem. 98, 207–214. 10.1016/j.nlm.2012.08.004 22922490

[B10] Beevers C. G. Wells T. T. McGeary J. E. (2009). The BDNF Val66Met polymorphism is associated with rumination in healthy adults. Emotion 9:579. 10.1037/a0016189 19653783 PMC2872140

[B11] Boone K. B. Ghaffarian S. Lesser I. M. Hill-Gutierrez E. G. Berman N. (1993). Wisconsin Card Sorting Test performance in healthy, older adults: relationship to age, sex, education, and IQ. J. Clin. Psychol. 49, 54–60. 10.1002/1097-4679(199301)49:1<54::AID-JCLP2270490108>3.0.CO2-6 8425935

[B12] Bremner J. D. Bolus R. Mayer E. A. (2007). Psychometric properties of the early trauma inventory–self report. J. Nerv. Ment. Dis. 195, 211–218. 10.1097/01.nmd.0000243824.84651.6c 17468680 PMC3229091

[B13] Buttelmann F. Karbach J. (2017). Development and plasticity of cognitive flexibility in early and middle childhood. Front. Psychol. 8:1040. 10.3389/fpsyg.2017.01040 28676784 PMC5476931

[B14] Caldwell W. McInnis O. A. McQuaid R. J. Liu G. Stead J. D. Anisman H.. (2013). The role of the Val66Met polymorphism of the brain derived neurotrophic factor gene in coping strategies relevant to depressive symptoms. PLoS ONE 8:e65547. 10.1371/journal.pone.0065547 23824678 PMC3688808

[B15] Cepeda N. J. Kramer A. F. Gonzalez de Sather J. (2001). Changes in executive control across the life span: examination of task-switching performance. Dev. Psychol. 37:715. 10.1037/0012-1649.37.5.715 11552766

[B16] Chamberlain S. R. Fineberg N. A. Blackwell A. D. Robbins T. W. Sahakian B. J. (2006). Motor inhibition and cognitive flexibility in obsessive-compulsive disorder and trichotillomania. Am. J. Psychiatry 163, 1282–1284. 10.1176/ajp.2006.163.7.1282 16816237

[B17] Chelune G. J. Baer R. A. (1986). Developmental norms for the Wisconsin card sorting test. J. Clin. Exp. Neuropsychol. 8, 219–228. 10.1080/01688638608401314 3722348

[B18] Clasen P. C. Wells T. T. Knopik V. S. McGeary J. E. Beevers C. G. (2011). 5-HTTLPR and BDNF Val66Met polymorphisms moderate effects of stress on rumination. Genes Brain Behav. 10, 740–746. 10.1111/j.1601-183X.2011.00715.x 21745335 PMC3401070

[B19] Cunha C. Brambilla R. Thomas K. L. (2010). A simple role for BDNF in learning and memory? Front. Mol. Neurosci. 3:1. 10.3389/neuro.02.001.2010 20162032 PMC2821174

[B20] Dajani D. R. Uddin L. Q. (2015). Demystifying cognitive flexibility: implications for clinical and developmental neuroscience. Trends Neurosci. 38, 571–578. 10.1016/j.tins.2015.07.003 26343956 PMC5414037

[B21] D'Amore D. E. Tracy B. A. Parikh V. (2013). Exogenous BDNF facilitates strategy set-shifting by modulating glutamate dynamics in the dorsal striatum. Neuropharmacology 75, 312–323. 10.1016/j.neuropharm.2013.07.033 23958449

[B22] Davis R. N. Nolen-Hoeksema S. (2000). Cognitive inflexibility among ruminators and nonruminators. Cogn. Ther. Res. 24, 699–711. 10.1023/A:1005591412406

[B23] de Waal M. M. Dekker J. J. Kikkert M. J. Kleinhesselink M. D. Goudriaan A. E. (2017). Gender differences in characteristics of physical and sexual victimization in patients with dual diagnosis: a cross-sectional study. BMC Psychiatry 17:270. 10.1186/s12888-017-1413-0 28743237 PMC5526321

[B24] Dick A. S. (2014). The development of cognitive flexibility beyond the preschool period: an investigation using a modified Flexible Item Selection Task. J. Exp. Child Psychol. 125, 13–34. 10.1016/j.jecp.2014.01.021 24814204

[B25] Egan M. F. Kojima M. Callicott J. H. Goldberg T. E. Kolachana B. S. Bertolino A.. (2003). The BDNF val66met polymorphism affects activity-dependent secretion of BDNF and human memory and hippocampal function. Cell 112, 257–269. 10.1016/S0092-8674(03)00035-7 12553913

[B26] Fox C. J. Mueller S. T. Gray H. M. Raber J. Piper B. J. (2013). Evaluation of a short-form of the Berg Card Sorting Test. PLoS ONE 8:e63885. 10.1371/journal.pone.0063885 23691107 PMC3653789

[B27] Gatt J. M. Nemeroff C. B. Dobson-Stone C. Paul R. H. Bryant R. A. Schofield P. R.. (2009). Interactions between BDNF Val66Met polymorphism and early life stress predict brain and arousal pathways to syndromal depression and anxiety. Mol. Psychiatry 14, 681–695. 10.1038/mp.2008.143 19153574

[B28] Han X. Wang W. Xue X. Shao F. Li N. (2011). Brief social isolation in early adolescence affects reversal learning and forebrain BDNF expression in adult rats. Brain Res. Bull. 86, 173–178. 10.1016/j.brainresbull.2011.07.008 21801814

[B29] Hayes A. F. (2013). Introduction to Mediation, Moderation, and Conditional Process Analysis: A Regression-Based Approach. New York, NY: Guilford Press.

[B30] Heaton R. K. Chelune G. J. Talley J. L. Kay G. C. Curtiss G. (1993). Wisconsin Card Sorting Test Manual. Psychological Assessment Resources, Odessa, FL.

[B31] Huizinga M. Dolan C. V. van der Molen M. W. (2006). Age-related change in executive function: developmental trends and a latent variable analysis. Neuropsychologia 44, 2017–2036. 10.1016/j.neuropsychologia.2006.01.010 16527316

[B32] Huizinga M. van der Molen M. W. (2007). Age-group differences in set-switching and set-maintenance on the Wisconsin Card Sorting Task. Dev. Neuropsychol. 31, 193–215. 10.1080/87565640701190817 17488216

[B33] Hurtubise J. L. Howland J. G. (2017). Effects of stress on behavioral flexibility in rodents. Neuroscience 345, 176–192. 10.1016/j.neuroscience.2016.04.007 27066767

[B34] Ionescu T. (2012). Exploring the nature of cognitive flexibility. New Ideas Psychol. 30, 190–200. 10.1016/j.newideapsych.2011.11.001

[B35] Kehagia A. A. Murray G. K. Robbins T. W. (2010). Learning and cognitive flexibility: frontostriatal function and monoamenergic modulation. Curr. Opin. Neurobiol. 20, 199–204. 10.1016/j.conb.2010.01.007 20167474

[B36] Kendler K. S. Thornton L. M. Prescott C. A. (2001). Gender differences in the rates of exposure to stressful life events and sensitivity to their depressogenic effects. Am. J. Psychiatry 158, 587–593. 10.1176/appi.ajp.158.4.587 11282693

[B37] Kovalchuk Y. Hanse E. Kafitz K. W. Konnerth A. (2002). Postsynaptic induction of BDNF-mediated long-term potentiation. Science 295, 1729–1734. 10.1126/science.1067766 11872844

[B38] Lamb Y. N. McKay N. S. Thompson C. S. Hamm J. P. Waldie K. E. Kirk I. J. (2015). Brain-derived neurotrophic factor Val66Met polymorphism, human memory, and synaptic neuroplasticity. Wiley Interdiscip. Rev. Cogn. Sci. 6, 97–108. 10.1002/wcs.1334 26263066

[B39] Lewin G. R. Barde Y. A. (1996). Physiology of the neurotrophins. Annu. Rev. Neurosci. 19, 289–317. 10.1146/annurev.ne.19.030196.001445 8833445

[B40] Lupien S. J. McEwen B. S. Gunnar M. R. Heim C. (2009). Effects of stress throughout the lifespan on the brain, behaviour and cognition. Nat. Rev. Neurosci. 10, 434–445. 10.1038/nrn2639 19401723

[B41] MacMillan H. L. Fleming J. E. Streiner D. L. Lin E. Boyle M. H. Jamieson E.. (2001). Childhood abuse and lifetime psychopathology in a community sample. Am. J. Psychiatry 158, 1878–1883. 10.1176/appi.ajp.158.11.1878 11691695

[B42] Maikovich-Fong A. K. Jaffee S. R. (2010). Sex differences in childhood sexual abuse characteristics and victims' emotional and behavioral problems: findings from a national sample of youth. Child Abuse Negl. 34, 429–437. 10.1016/j.chiabu.2009.10.006 20400178 PMC2913705

[B43] McEwen B. S. Morrison J. H. (2013). The brain on stress: vulnerability and plasticity of the prefrontal cortex over the life course. Neuron 79, 16–29. 10.1016/j.neuron.2013.06.028 23849196 PMC3753223

[B44] Meng X. D'Arcy C. (2016). Gender moderates the relationship between childhood abuse and internalizing and substance use disorders later in life: a cross-sectional analysis. BMC Psychiatry 16:401. 10.1186/s12888-016-1071-7 27846829 PMC5111209

[B45] Mueller S. T. Piper B. J. (2014). The psychology experiment building language (PEBL) and PEBL test battery. J. Neurosci. Methods 222, 250–259. 10.1016/j.jneumeth.2013.10.024 24269254 PMC3897935

[B46] Pezawas L. Verchinski B. A. Mattay V. S. Callicott J. H. Kolachana B. S. Straub R. E.. (2004). The brain-derived neurotrophic factor val66met polymorphism and variation in human cortical morphology. J. Neurosci. 24, 10099–10102. 10.1523/JNEUROSCI.2680-04.2004 15537879 PMC6730170

[B47] Rybakowski J. K. Borkowska A. Czerski P. M. Skibinska M. Hauser J. (2003). Polymorphism of the brain-derived neurotrophic factor gene and performance on a cognitive prefrontal test in bipolar patients. Bipolar Disord. 5, 468–472. 10.1046/j.1399-5618.2003.00071.x 14636373

[B48] Rybakowski J. K. Borkowska A. Skibinska M. Szczepankiewicz A. Kapelski P. Leszczynska-Rodziewicz A.. (2006). Prefrontal cognition in schizophrenia and bipolar illness in relation to Val66Met polymorphism of the brain-derived neurotrophic factor gene. Psychiatry Clin. Neurosci. 60, 70–76. 10.1111/j.1440-1819.2006.01462.x 16472361

[B49] Sakata K. Martinowich K. Woo N. H. Schloesser R. J. Jimenez D. V. Ji Y.. (2013). Role of activity-dependent BDNF expression in hippocampal–prefrontal cortical regulation of behavioral perseverance. Proc. Natl. Acad. Sci. U.S.A. 110, 15103–15108. 10.1073/pnas.1222872110 23980178 PMC3773762

[B50] Savitz J. Solms M. Ramesar R. (2006). The molecular genetics of cognition: dopamine, COMT and BDNF. Genes Brain Behav. 5, 311–328. 10.1111/j.1601-183X.2005.00163.x 16716201

[B51] Spann M. N. Mayes L. C. Kalmar J. H. Guiney J. Womer F. Y. Pittman B.. (2012). Childhood abuse and neglect and cognitive flexibility in adolescents. Child Neuropsychol. 18, 182–189. 10.1080/09297049.2011.595400 21942637 PMC3326262

[B52] Spielberger C. D. Gorsuch R. L. Lushene R. Vagg P. R. Jacobs G. A. (1983). Manual for the State-Trait Anxiety Inventory. Palo Alto, CA: Consulting Psychologists Press.

[B53] Stoltenborgh M. Van Ijzendoorn M. H. Euser E. M. Bakermans-Kranenburg M. J. (2011). A global perspective on child sexual abuse: Meta-analysis of prevalence around the world. Child maltreat. 16, 79–101. 10.1177/1077559511403920 21511741

[B54] Thoma P. Wiebel B. Daum I. (2007). Response inhibition and cognitive flexibility in schizophrenia with and without comorbid substance use disorder. Schizophr. Res. 92, 168–180. 10.1016/j.schres.2007.02.004 17399952

[B55] Thompson M. P. Kingree J. B. Desai S. (2004). Gender differences in long-term health consequences of physical abuse of children: data from a nationally representative survey. Am. J. Public Health 94, 599–604. 10.2105/AJPH.94.4.599 15054012 PMC1448305

[B56] Trivedi M. H. Greer T. L. (2014). Cognitive dysfunction in unipolar depression: implications for treatment. J. Affect. Disord. 152–154, 19–27. 10.1016/j.jad.2013.09.012 24215896

[B57] Watson D. Clark L. A. Tellegen A. (1988). Development and validation of brief measures of positive and negative affect: the PANAS scales. J. Pers. Soc. Psychol. 54, 1063–1070. 10.1037/0022-3514.54.6.1063 3397865

[B58] Zelzo P. D. (2006). The dimensional change card sort (DCCS): a method of assessing executive function in children. Nat. Protoc. 1, 297–301. 10.1038/nprot.2006.46 17406248

